# Defining patient‐centered amyloid PET thresholds for the onset of tauopathy in Alzheimer's disease

**DOI:** 10.1002/alz.71064

**Published:** 2026-01-04

**Authors:** Zeyu Zhu, Anna Steward, Amir Dehsarvi, Sebastian N. Roemer‐Cassiano, Anna Dewenter, Davina Biel, Fabian Hirsch, Lukas Frontzkowski, Julia Pescoller, Madleen Klonowski, Johannes Gnörich, Michael J. Pontecorvo, Sergey Shcherbinin, Michael Schöll, Rachel Buckley, Rik Ossenkoppele, Fang Xie, Tengfei Guo, Alzheimer's Disease Neuroimaging Initiative, Günter Höglinger, Matthias Brendel, Nicolai Franzmeier

**Affiliations:** ^1^ Institute for Stroke and Dementia Research (ISD) University Hospital LMU Munich Munich Germany; ^2^ Department of Neurology University Hospital LMU Munich Munich Germany; ^3^ Max Planck School of Cognition Leipzig Germany; ^4^ Department of Nuclear Medicine University Hospital LMU Munich Munich Germany; ^5^ Eli Lilly and Company Indianapolis Indiana USA; ^6^ Department of Psychiatry and Neurochemistry The Sahlgrenska Academy Institute of Neuroscience and Physiology University of Gothenburg Gothenburg Sweden; ^7^ Wallenberg Centre for Molecular and Translational Medicine and the Department of Psychiatry and Neurochemistry University of Gothenburg Gothenburg Sweden; ^8^ Dementia Research Centre Queen Square Institute of Neurology University College London London UK; ^9^ Massachusetts General Hospital Harvard Medical School Boston Massachusetts USA; ^10^ Department of Clinical Sciences Malmö Clinical Memory Research Unit Faculty of Medicine Lund University Lund Sweden; ^11^ Alzheimer Center Amsterdam Neurology Vrije Universiteit Amsterdam Amsterdam the Netherlands; ^12^ Amsterdam Neuroscience Neurodegeneration Amsterdam the Netherlands; ^13^ Department of Nuclear Medicine & PET Center Huashan Hospital Fudan University Shanghai China; ^14^ Institute of Neurological and Psychiatric Disorders Shenzhen Bay Laboratory Shenzhen China; ^15^ Institute of Biomedical Engineering Peking University Shenzhen Graduate School Shenzhen China; ^16^ German Center for Neurodegenerative Diseases (DZNE) Munich Germany; ^17^ Munich Cluster for Systems Neurology (SyNergy) Munich Germany

**Keywords:** amyloid positron emission tomography, amyloid positron emission tomography thresholds, tauopathy, tau positron emission tomography

## Abstract

**INTRODUCTION:**

Amyloid‐induced tauopathy drives clinical decline in Alzheimer's disease (AD). Because age and sex shape tau trajectories, defining patient‐centered amyloid thresholds for tauopathy onset could facilitate pre‐tauopathy AD identification and aid treatment decisions and prognosis.

**METHODS:**

By including two samples (Alzheimer's Disease Neuroimaging Initiative [ADNI, *n* = 301]; and 18F‐AV‐1451‐A05 [A05, *n* = 143]), we explored whether age and sex affect tauopathy transition and determined patient‐centered amyloid positron emission tomography (PET) thresholds that mark tauopathy onset.

**RESULTS:**

We found a consistent amyloid PET × age interaction on global tau PET increase in men (ADNI/A05: *p* = 0.0078/0.018), with younger men showing faster amyloid‐associated tau accumulation. We then established patient‐centered, amyloid PET–inferred tauopathy transition cut‐offs. Women reached this transition at lower amyloid PET levels, and these cutoffs predicted both earlier onset and accelerated cognitive decline (*p* < 0.001).

**DISCUSSION:**

This study highlights the effect of age and sex on the amyloid‐to‐tauopathy transition, establishes patient‐centered amyloid PET thresholds for tauopathy onset, and links these thresholds to accelerated cognitive decline.

**Highlights:**

- Younger age is related to faster amyloid‐related tau accumulation in men.
- We defined a series of amyloid positron emission tomography (PET) thresholds to enable patient‐centered inference of amyloid‐related tauopathy.
- Crossing the amyloid PET–defined tauopathy phase is associated with more progressive tau deposition and cognitive decline.

## 1 BACKGROUND

According to the amyloid cascade of Alzheimer's disease (AD),^1^, ^2^ amyloid beta (Aβ) deposition triggers tau aggregation, ensuing neurodegeneration and cognitive decline.^3^, ^4^, ^5^ Thus, anti‐Aβ drugs have been a key focus of therapeutic development to attenuate AD progression and cognitive decline.^1^, ^6^, ^7^, ^8^, ^9^, ^10^, ^11^ Indeed, the development of anti‐Aβ antibodies has been a key therapeutic success in AD, showing that Aβ‐plaque reduction back to normal levels can be therapeutically achieved.^12^, ^13^, ^14^, ^15^ However, the clinical efficacy of anti‐Aβ treatments has varied substantially.^15^, ^16^ Here, removal of Aβ with lecanemab or donanemab has yielded up to 35% delay of cognitive decline,^8^, ^11^, ^17^ while gantenerumab showed no clinical benefit^10^ and aducanumab yielded inconsistent results across two trials.^18^ Despite the diverse biological properties of anti‐Aβ antibodies, including amyloid binding, immune activation, and other functional characteristics, the clinical trial design and timing of Aβ removal may influence clinical efficacy.^16^ This is, in particular, supported by a *post mortem* study revealing sparse Aβ plaques with advanced neurofibrillary tangle pathology (Braak stage V) in an aducanumab‐treated patient who progressed from moderate to end‐stage dementia, suggesting limited effects of Aβ clearance at advanced tau and clinical stages.^19^ Thus, defining therapeutic windows for Aβ removal may represent an important step toward optimizing clinical efficacy.

The TRAILBLAZER‐ALZ 2 phase 3 trial of donanemab demonstrated that patients with a low/medium tau burden on tau positron emission tomography (PET) showed greater clinical benefit over 18 months than patients with a high tau burden.^11^ This result is expected, given that Aβ drives tau aggregation, which in turn drives neurodegeneration and cognitive decline;^3^, ^4^, ^20^, ^21^, ^22^ hence, therapeutic removal of Aβ should ideally take place prior to tauopathy onset to prevent neurodegeneration and cognitive decline. In other words, the evolving transition from amyloidosis to tauopathy may be a limiting factor for the clinical efficacy of anti‐Aβ treatments in AD.^13^ Therefore, establishing Aβ biomarker cut‐offs that indicate the amyloidosis to tauopathy transition, rather than just the presence of Aβ,^23^ may help screen patients and better define optimal treatment windows for anti‐Aβ drugs. Previous studies suggested 68 Centiloids as the cut‐off for greater tau accumulation in flortaucipir.^24^ However, the well‐documented heterogeneity in the Aβ–tau axis wasn't taken into account, in which female sex and younger age are consistently linked to faster tau accumulation in AD, potentially leading to earlier tauopathy onset and reduced anti‐Aβ treatment efficacy.^25^, ^26^, ^27^, ^28^, ^29^ This is supported by results from the Clarity AD phase 3 trial of lecanemab, showing attenuated clinical efficacy of anti‐Aβ treatment in younger versus older patients and in women versus men.^14^ A combination of amyloid PET and tau PET is a viable option for staging amyloidosis and tauopathy, but this approach seems unfeasible in real‐world clinical practice, in which dual PET scanning comes with substantial financial and logistic burden. Fluid biomarkers of tau pathophysiology (e.g., phosphorylated tau [p‐tau]_217_) most likely capture secreted hyperphosphorylated tau rather than tangles and are not yet established to stage fibrillar tau, which triggers neurodegeneration.^30^, ^31^ An alternative option is proxy staging of Aβ‐associated tauopathy onset, that is, by defining associations between amyloid PET and tau PET in larger cohorts and extrapolating amyloid PET–based cut‐offs that signal the transition from amyloidosis to tauopathy, considering age and sex as modulating factors.

Therefore, our major aims were to (1) recapitulate how age and sex modulate Aβ‐related tau accumulation, (2) define age‐ and sex‐stratified amyloid PET thresholds that signal the onset of significant neocortical tau accumulation, and (3) assess whether more advanced amyloid PET–inferred tauopathy stages translate into faster cognitive decline.

## 2 METHODS

### 2.1 Participants

We included 301 Alzheimer's Disease Neuroimaging Initiative (ADNI) participants, with baseline structural magnetic resonance imaging (MRI), amyloid PET ([^18^F] Florbetapir/Florbetaben: *n* = 218/83), and at least two [^18^F]Flortaucipir tau PET scans and neuropsychological examination. ADNI investigators diagnosed subjects as cognitively normal (CN; Mini‐Mental State Examination [MMSE] ≥ 24, Clinical Dementia Rating [CDR] = 0, non‐depressed), mildly cognitively impaired (MCI; MMSE ≥ 24, CDR = 0.5, objective memory impairment on the education‐adjusted Wechsler Memory Scale II, preserved activities of daily living), or demented (MMSE = 20–26, CDR ≥ 0.5, National Institute for Neurological and Communicative Disorders and Stroke/Alzheimer's Disease and Related Disorders Association criteria for probable AD). Further, we included 143 participants from the 18F‐AV‐1451‐A05 [A05] phase 2/3 study (NCT02116010), with baseline structural MRI, [^18^F]Florbetapir amyloid PET, and at least two [^18^F]Flortaucipir tau PET scans and clinical assessments. Participants were classified by the A05 study core as CN (MMSE ≥ 29, no history of cognitive impairment), MCI (24 ≤ MMSE ≤ 29, meeting the 2011 National Institute on Aging–Alzheimer's Association [NIA‐AA] criteria for MCI)^32^ and dementia (10 < MMSE < 24, meeting the 2011 NIA‐AA criteria for possible or probable AD).^33^ Global amyloid PET standardized uptake value ratios (SUVRs) were used to determine amyloid status (i.e., Aβ–‐/+) using pre‐established thresholds (i.e., Aβ+ > 1.11/1.08 for Florbetapir/Florbetaben)^34^, ^35^ and were transformed to Centiloid to harmonize across amyloid PET tracers within each sample.^36^, ^37^, ^38^, ^39^ Aβ– subjects with a diagnosis other than CN were excluded, to avoid comorbid non‐AD pathology driving cognitive impairment. Participants were considered apolipoprotein E (*APOE*) ε4 risk allele carriers if they had at least one ε4 allele. Ethical approval was obtained by ADNI and A05 investigators at each site, and all subjects provided written informed consent.

**RESEARCH IN CONTEXT**

1. **Systematic review**: Advanced tauopathy limits the clinical efficacy of anti‐amyloid treatment, suggesting that treatment should be initiated at pre‐tauopathy stages. Although previous studies suggested that amyloid positron emission tomography (PET) can predict group‐level tau accumulation rates and stage disease, the well‐documented heterogeneity in the amyloid beta–tau axis has not yet been considered.
2. **Interpretation**: In two independent samples, we show that younger age was associated with faster amyloid‐related tau accumulation rates in men, while women transitioned to tauopathy at lower amyloid levels. Based on this, we defined patient‐centered amyloid thresholds (i.e., considering age and sex) to mark the transition to tauopathy, which successfully captured progressive tau aggregation and associated cognitive decline. Our approach complements established binary amyloid PET–positivity approaches by implementing patient‐centered inference of amyloid‐associated tauopathy stages, which may inform personalized Alzheimer's disease treatment strategies.
3. **Future directions**: Future work should incorporate clinical trial data to validate and quantify clinical efficacy of anti‐amyloid treatments before and after crossing amyloid‐inferred tauopathy thresholds.

### 2.2 Neuroimaging acquisition and processing

For ADNI, T1‐weighted structural MRI was collected using 3T scanners. PET data were recorded post‐intravenous injection of [18F]‐labeled tracers (Flortaucipir: 6 × 5 minute frames, 75–105 minutes post‐injection; Florbetapir: 4 × 5 minute frames, 50–70 minutes post‐injection; and Florbetaben: 4 × 5 minute frames, 90–110 minutes post‐injection). Using the CAT12 toolbox,^40^ T1‐weighted MRI scans were segmented and non‐linearly warped to Montreal Neurological Institute (MNI) space.^40^ Static PET images were obtained by realigning and averaging dynamic PET images and subsequently registered to the T1‐weighted MRI. The CAT12‐derived non‐linear normalization parameters were used to warp the reference regions (i.e., inferior cerebellar gray for [18F]‐Flortaucipir^41^ and whole cerebellum for [18F]‐Florbetapir/Florbetaben^42^) and the 200 region of interest (ROI) Schaefer atlas from MNI to T1‐native space.^43^ Subject‐specific gray matter was further used to mask ROIs and then applied to T1‐registered PET images to calculate SUVRs. [18F]‐Florbetapir/Florbetaben SUVRs were converted to Centiloids based on equations provided by ADNI.

For A05, structural T1‐weighted MRI data were collected using 1.5 or 3T scanners. PET data were acquired post‐intravenous injection of [18F]‐ Flortaucipir (4 × 5 minute frames, ≈ 80 minutes post‐injection) and [18F]‐Florbetapir (2 × 5 minute frames, ≈ 50 minutes post‐injection). Data acquisition and processing (performed by Avid investigators) have been described in detail previously.^44^ Specifically, native‐space PET images were rigidly co‐registered to T1‐weighted MRI, then spatially normalized to MNI space with FSL's FNIRT and intensity normalized to the whole cerebellum for amyloid PET and inferior cerebellar gray for tau PET. PET images were parcellated using the Schaefer 200 ROI atlas for further SUVR calculation, and global amyloid PET SUVRs were transformed to Centiloid.^43^ Linear mixed models adjusted for random slope and intercept were used to determine the tau PET annual rate of change (ROC) for each Schaefer ROI, as well as a global cortical and temporal meta ROI in both samples, as established previously.^45^, ^46^, ^47^

### 2.3 Statistics

For aim 1, we tested whether younger age is associated with faster Aβ‐related tau accumulation and whether this effect was consistent across both sexes. Because ordinary least squares regression is sensitive to outliers, we used robust regression to ensure that our conclusions were not driven by extreme values or skewed data.^48^, ^49^, ^50^, ^51^, ^52^ We then assessed the age by amyloid PET interaction on global and temporal meta tau PET change rates, stratified by sex and adjusted for *APOE* ε4 status. Equivalent regression models were determined for tau PET change rates of 200 cortical ROIs of the Schaefer atlas,^53^ to map amyloid PET by age interaction patterns. We further used bootstrapping to estimate slope distributions and 95% confidence intervals (CIs) for the effect of amyloid PET on tau PET change rates, stratified by age percentiles for each sex, to confirm that Aβ’s impact on tau accumulation was greater at younger ages and to assess whether this effect differed between sexes.

For aim 2, we determined age‐ and sex‐specific amyloid PET cut‐offs at which individuals transition to tauopathy. We defined the onset of tauopathy by determining the *z* score of temporal meta tau PET using the mean and standard deviation of the CN Aβ– as a reference group. Three severity stages of tauopathy were defined as mild, moderate, and severe tauopathy, when surpassing *z* score thresholds of 2, 2.5, and 3, respectively. For this step, ADNI and A05 were pooled to increase statistical power and generalizability, while adjusting all analyses for cohort as a covariate. Using robust regression with 1000 bootstrapped iterations, we estimated slope associations between amyloid PET and temporal meta tau PET change rates stratified by age and sex groups (i.e., ≤ 65/ > 65–80/ > 80), adjusting for *APOE* ε4 and cohort. Using these slope estimates, cut‐offs for transitioning to mild/moderate/severe tauopathy were defined as amyloid PET levels at which the predicted 95% CI of the slope estimate surpassed the *z* score threshold of 2/2.5/3.

For aim 3, we tested whether more advanced amyloid PET–inferred tauopathy phases were linked to faster cognitive decline. We used the age‐ and sex‐specific amyloid PET thresholds defined for aim 2, and classified individuals as falling below or above a given threshold (> mild/ > moderate/ > severe) or within gradual categories (i.e., < mild, mild–moderate, moderate–severe, > severe). Cognitive change rates were determined using longitudinal MMSE and Alzheimer's Disease Assessment Scale Cognitive subscale (ADAS‐Cog_11_) data using linear mixed models adjusted for random slope and intercept. To model non‐linear cognitive trajectories, we estimated onset timing and acceleration of cognitive decline using the Python‐based Leaspy package.^54^ Specifically, we used 10‐fold cross‐validation to increase generalizability. Within each training fold, the group‐level trajectory was estimated using the Markov chain Monte Carlo—stochastic approximation expectation maximization (mcmc_saem) algorithm.^55^ After fitting the population model, we personalized it in the corresponding test fold by the gradient‐based algorithm (L‐BFGS),^56^ parameterizing each participant's temporal variability with a time‐shift and an acceleration factor. These parameters quantified each subject's relative onset and progression rate versus the population trajectory. Using analyses of covariance (ANCOVAs) and post hoc Tukey tests, we then determined differences in cognitive change rates, as well as cognitive decline onset timing and acceleration between amyloid PET–inferred tauopathy groups, controlling for age, sex, education, and cohort. Analyses were performed in R 4.4.1 and Python 3.10.0.

### 2.4 Data availability

ADNI data are available online upon registration (https://ida.loni.usc.edu/). A05 data can be obtained upon request and completion of a data sharing agreement with the principal investigators at Avid Radiopharmaceuticals, a subsidiary of Eli Lilly and Company.

## 3 RESULTS

### 3.1 Sample characteristics

We included 301 ADNI (i.e., 109 CN Aβ–, 97 CN Aβ+, 64 MCI Aβ+, and 31 dementia Aβ+) and 143 A05 (i.e., 41 CN Aβ–, 3 CN Aβ+, 58 MCI Aβ+, and 41 dementia Aβ+) participants with baseline amyloid PET, longitudinal tau PET, and cognitive assessments. As expected, tau PET SUVRs and change rates increased with clinical disease severity across the AD spectrum (Figure S1 in supporting information). Average tau PET follow‐up was 2.86 ± 1.34/1.21 ± 0.37 years in ADNI/A05. Detailed sample characteristics by sex and age groups are reported in Table 1, and cohort differences are shown in Table S1 in supporting information.

**TABLE 1:** Sample characteristics by sex and age group.

	Male				Female			
ADNI	≤ 65 *n* = 7	65–80 *n* = 97	> 80 *n* = 36	*p* value	≤ 65 *n* = 21	65–80 *n* = 116	> 80 *n* = 24	*p* value
Global amyloid PET (Centiloid, M/SD)	73.36 (56.01)	42.99 (40.61)	74.72 (41.33)	0.004	34.28 (38.24)	48.61 (41.13)	51.45 (39.03)	0.3
Tau PET follow‐up time in years (M/SD)	2.178 (1.162)	2.749 (1.400)	2.487 (1.366)	0.4	3.225 (1.057)	3.170 (1.273)	2.228 (1.265)	0.006
Global tau PET ROC (M/SD)	0.0554 (0.0613)	0.0048 (0.0158)	0.0062 (0.0107)	0.13	0.0112 (0.0195)	0.0080 (0.0140)	0.0063 (0.0097)	0.6
Temporal tau PET ROC (M/SD)	0.0586 (0.0576)	0.0136 (0.0216)	0.0197 (0.0194)	0.079	0.0182 (0.0271)	0.0172 (0.0231)	0.0185 (0.0209)	>0.9
*APOE* ε4_status (pos/neg)	5/2	46/51	18/18	0.5	15/6	55/61	8/16	0.032
Diagnosis (CN/MCI/dementia)	2/2/3	63/24/10	17/11/8	0.045	14/7/0	95/16/5	15/4/5	0.009
Baseline MMSE (M/SD)	25.57 (3.552)	28.20 (2.230)	27.00 (2.414)	0.027	28.52 (1.750)	28.59 (2.526)	27.04 (3.747)	0.2
Baseline ADAS‐Cog_11_ (M/SD)	13.57 (9.110)	7.563 (4.797)	10.15 (6.171)	0.051	6.286 (3.697)	5.690 (5.259)	9.082 (7.331)	0.11

	**Male**				**Female**			
**A05**	**≤ 65** *n* = 15	**65–80** *n* = 44	**> 80** *n* = 21	***p* value**	**≤ 65** *n* = 15	**65–80** *n* = 36	**> 80** *n* = 12	***p* value**
Global amyloid PET (Centiloid, M/SD)	23.05 (43.36)	65.47 (46.91)	71.36 (53.80)	0.006	63.84 (57.77)	62.31 (55.77)	64.78 (49.04)	>0.9
Tau PET follow‐up time in years (M/SD)	1.250 (0.3660)	1.159 (0.3778)	1.179 (0.3803)	0.7	1.200 (0.3803)	1.271 (0.3504)	1.188 (0.3862)	0.7
Global tau PET ROC (M/SD)	0.0181 (0.0303)	0.0148 (0.0164)	0.0106 (0.0107)	0.4	0.0342 (0.0310)	0.0234 (0.0213)	0.0180 (0.0151)	0.2
Temporal tau PET ROC (M/SD)	0.0119 (0.0199)	0.0126 (0.0105)	0.0102 (0.0070)	0.6	0.0232 (0.0196)	0.0193 (0.0155)	0.0153 (0.0108)	0.4
*APOE* ε4 status (pos/neg)	6/9	21/23	6/15	0.4	7/8	20/16	6/6	0.8
Diagnosis (CN/MCI/dementia)	10/2/3	11/23/10	5/8/8	0.020	5/6/4	9/17/10	4/2/6	0.4
Baseline MMSE (M/SD)	27.93 (2.549)	26.18 (4.025)	25.90 (3.161)	0.077	26.93 (4.008)	26.53 (3.047)	23.67 (4.271)	0.11
Baseline ADAS‐Cog_11_ (M/SD)	10.33 (8.304)	11.91 (7.389)	14.10 (5.603)	0.2	12.93 (10.12)	12.69 (7.660)	15.75 (8.895)	0.6

Notes: Analysis of variance tests were used for continuous variables, and Fisher exact tests were used for categorical variables in group comparisons, with a two‐sided alpha level of 0.05.

Abbreviations: A05, 18F‐AV‐1451‐A05; ADAS‐Cog_11_, Alzheimer's Disease Assessment Scale Cognitive subscale; ADNI, Alzheimer's Disease Neuroimaging Initiative; *APOE*, apolipoprotein E; CN, cognitively normal; MCI, mild cognitive impairment; MMSE, Mini‐Mental State Examination; PET, positron emission tomography; ROC, receiver operating characteristic; SD, standard deviation.

### 3.2 Younger age is related to faster Aβ‐related tau accumulation in men

**FIGURE 1:**
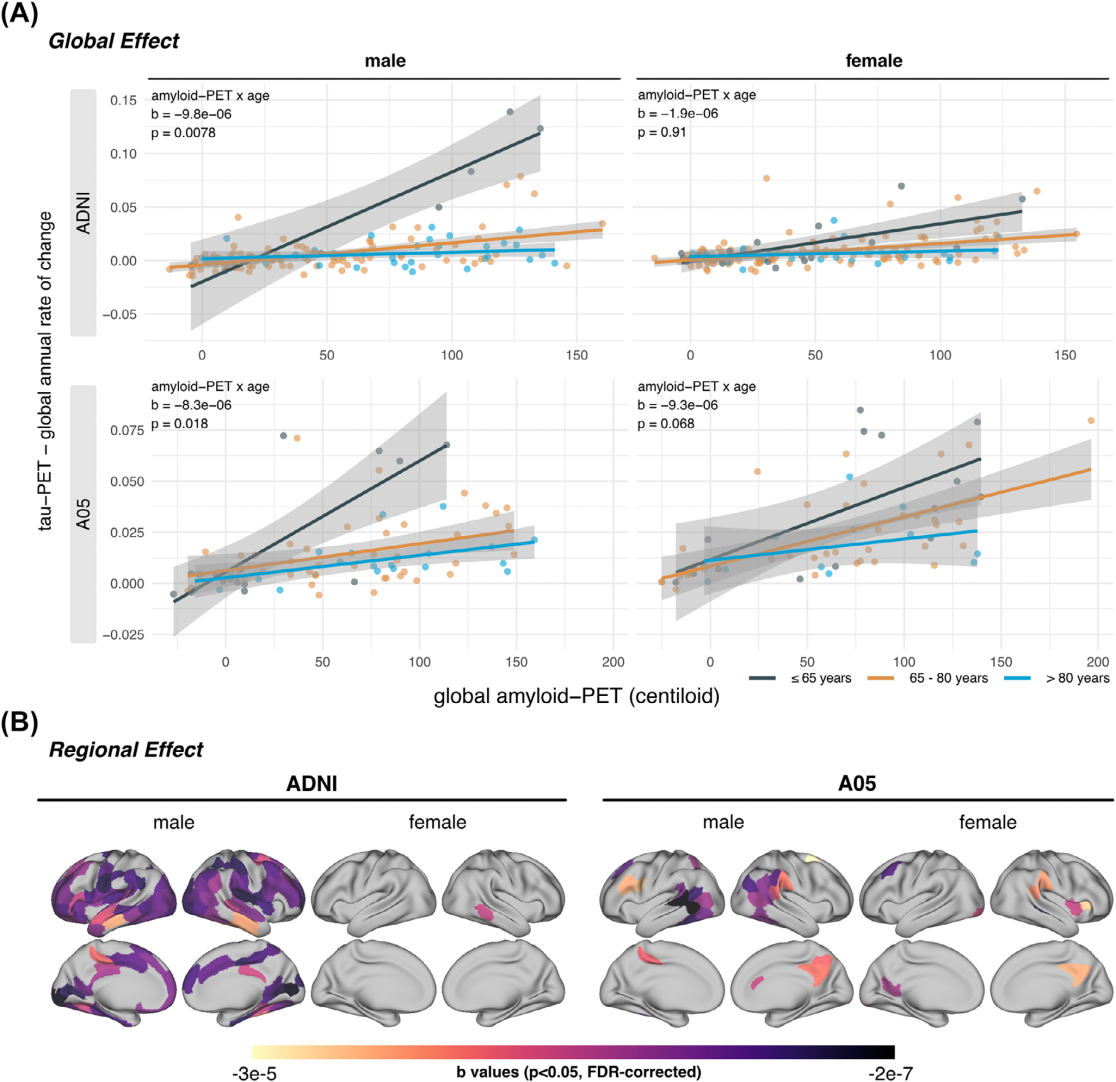
Effects of age on Aβ‐related tau accumulation by sex. Scatterplots showing the interaction between global amyloid PET (Centiloid) and age on global tau PET annual change rates stratified by sex (A–D). Regional mapping of the age × amyloid PET interaction (E, F). Statistics were determined based on robust regression, adjusting for *APOE* ε4 carrier status. *p* values for panels (A)–(D) were Bonferroni corrected (*p* < 0.05), and regional analyses in panels (E) and (F) were FDR corrected (*p* < 0.05) within each cohort and sex. A05, 18F‐AV‐1451‐A05; Aβ, amyloid beta; ADNI, Alzheimer's Disease Neuroimaging Initiative; *APOE*, apolipoprotein E; FDR, false discovery rate; PET, positron emission tomography.

First, we aimed to confirm that younger age is related to faster Aβ‐related tau accumulation,^28^, ^29^ and determined whether this effect was present across both sexes. To this end, we used sex‐stratified robust regression and calculated the age by amyloid PET interaction on global tau PET change rates. We found that younger age was associated with faster Aβ‐related tau accumulation in men (ADNI: *b* = −9.8×10^−6^, *P*_Bonferroni‐corrected _= 0.0078; A05: *b* = −8.3×10^−6^, *P*_Bonferroni‐corrected _= 0.018; Figure 1A, left panel). The association persisted after additional adjustment for cognitive status (Table S2 in supporting information). In contrast, no such association was observed in female ADNI participants (*b* = −1.9×10^−6^, *P*_Bonferroni‐corrected _= 0.91; Figure 1A, upper‐right panel), whereas female A05 participants showed a comparable trend to men (*b* = −9.3×10^−6^, *P*_Bonferroni‐corrected _= 0.068; Figure 1A, lower‐right panel). Results were largely consistent in the temporal meta‐ROI that younger men were associated with faster temporal meta‐tau PET change rates, while this association was weaker/absent in women (Figure S2 in supporting information). Regional mapping of the amyloid PET by age interaction revealed widespread effects in male ADNI participants (110 regions reaching significance) across temporal, occipital, parietal, and frontal regions, and a similar though less extensive pattern in male A05 participants (27 regions reaching significance) across temporal, occipital, and parietal regions. In contrast, effects were limited in women (ADNI/A05: 1/9 regions reaching a statistically significant effect; Figures 1B and S3 in supporting information). For further validation, we compared the sex‐specific effect of amyloid PET on temporal meta‐tau PET change rates between nine adjacent percentile‐defined age groups (10th–90th), using bootstrapped robust regression estimates within each age group. Consistent with the previous analyses, we found that the effect of amyloid PET on tau PET change rates was consistent across age percentiles in women but strengthened in men at younger ages (Figure S4 in supporting information). Importantly, men and women showed no differences in baseline global and temporal meta‐ROI tau PET levels at the same age group (i.e., < 65/65–80/ > 80 years, *t* tests all *p* > 0.05), suggesting that the acceleration of Aβ‐related tau accumulation at younger age in men is not explained by different baseline tau PET levels. Overall, these results suggest that younger age is consistently associated with faster Aβ‐related tau accumulation in men.

### 3.3 Defining age‐ and sex‐stratified amyloid PET thresholds for the onset of tauopathy

**FIGURE 2:**
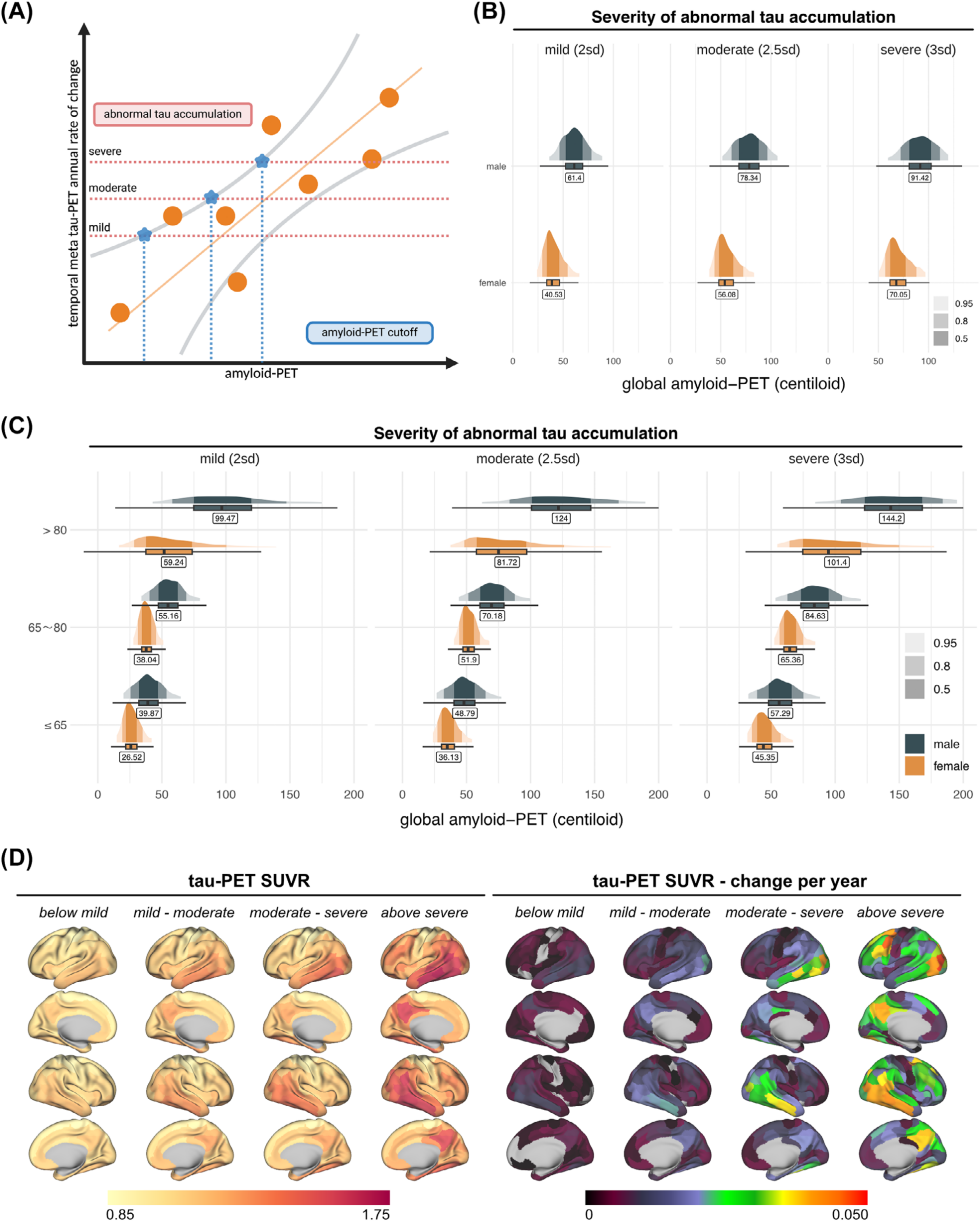
Detection of amyloid PET thresholds at which patients enter the tauopathy phase. A, Graphic illustration of threshold detection created with BioRender.com. Yellow dots represent individual participants. Gray curves depict the 95% confidence interval of the predicted temporal meta tau PET ROC, estimated from regression models using baseline amyloid PET and temporal meta tau PET ROC. Red horizontal dashed lines denote the predefined cutoff for abnormal temporal meta tau PET ROC. Blue pentagons indicate the amyloid PET threshold, defined as the amyloid PET value at which the upper 95% CI first crosses this cutoff. B, Amyloid PET values for males and females entering different tauopathy phases. C, Amyloid PET values for males and females entering different tauopathy phases stratified by age group. D, Baseline tau PET SUVR and longitudinal tau PET annual rate of change among different amyloid‐defined tauopathy phases. CI, confidence interval; PET, positron emission tomography; ROC, receiver operating characteristic; SUVR, standardized uptake value ratio.

Second, we defined amyloid PET thresholds signaling the transition from amyloidosis to tauopathy, determined as the minimum Centiloid level at which annual temporal meta‐tau PET change rates exceeded the *z* score thresholds for mild/moderate/severe tauopathy (Figure 2A). We found that women transition to tauopathy at lower amyloid PET levels than men (Figure 2B), where Centiloid thresholds for mild tauopathy were 40.53 (95% CI: [24.00; 66.17]) in women versus 61.40 (95% CI: [36.17; 88.83]) in men. For moderate tauopathy, the thresholds were 56.08 (95% CI: [38.75; 83.17]) in women and 78.34 (95% CI: [51.58; 106.42]) in men. For severe tauopathy, the thresholds were 70.05 (95% CI: [50.67; 96.75]) in women and 91.42 (95% CI: [59.63; 119.33]) in men. Because younger men showed faster Aβ‐related tau accumulation, we further stratified amyloid PET thresholds by age, where women consistently showed lower Centiloid thresholds for transitioning to tauopathy across all age groups (Figure 2C). When mapping tau PET by amyloid PET–inferred tauopathy groups, we found higher baseline tau PET SUVRs and faster change rates with increasing amyloid PET–inferred tauopathy stages (Figure 2D). Together, these findings suggest that age‐ and sex‐specific amyloid PET thresholds for tauopathy successfully capture increasing tau severity levels.

### 3.4 Advanced amyloid PET–inferred tauopathy phases translate into more progressive cognitive decline

**FIGURE 3:**
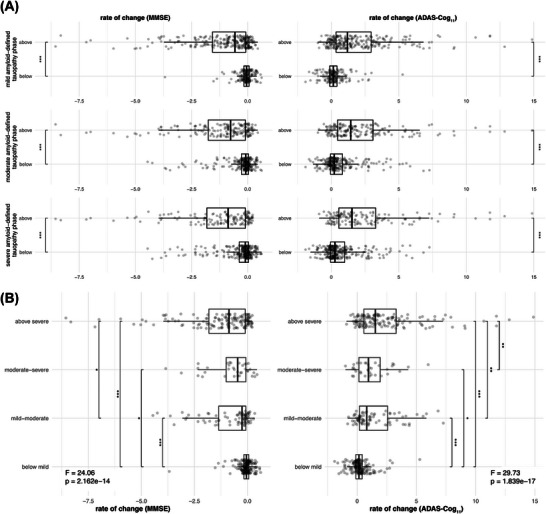
Linear cognitive decline speed per tauopathy phase status defined by baseline amyloid PET level. A, The annual rate of change between individuals with amyloid PET levels below and above calculated amyloid PET thresholds for various tauopathy phases was compared using ANCOVA tests. B, Participants were further divided into four groups based on different amyloid PET thresholds (i.e., thresholds for mild/moderate/severe tauopathy phases), followed by ANCOVA and Tukey tests to detect differences in cognitive decline rate across each group. Sex, age, education, and cohort were controlled. ^*^ *p* < 0.05, ^**^ *p* < 0.01, ^***^ *p* < 0.001. ADAS‐Cog_11_, Alzheimer's Disease Assessment Scale Cognitive subscale; ANCOVA, analysis of covariance; MMSE, Min‐Mental State Examination; PET, positron emission tomography.

**FIGURE 4:**
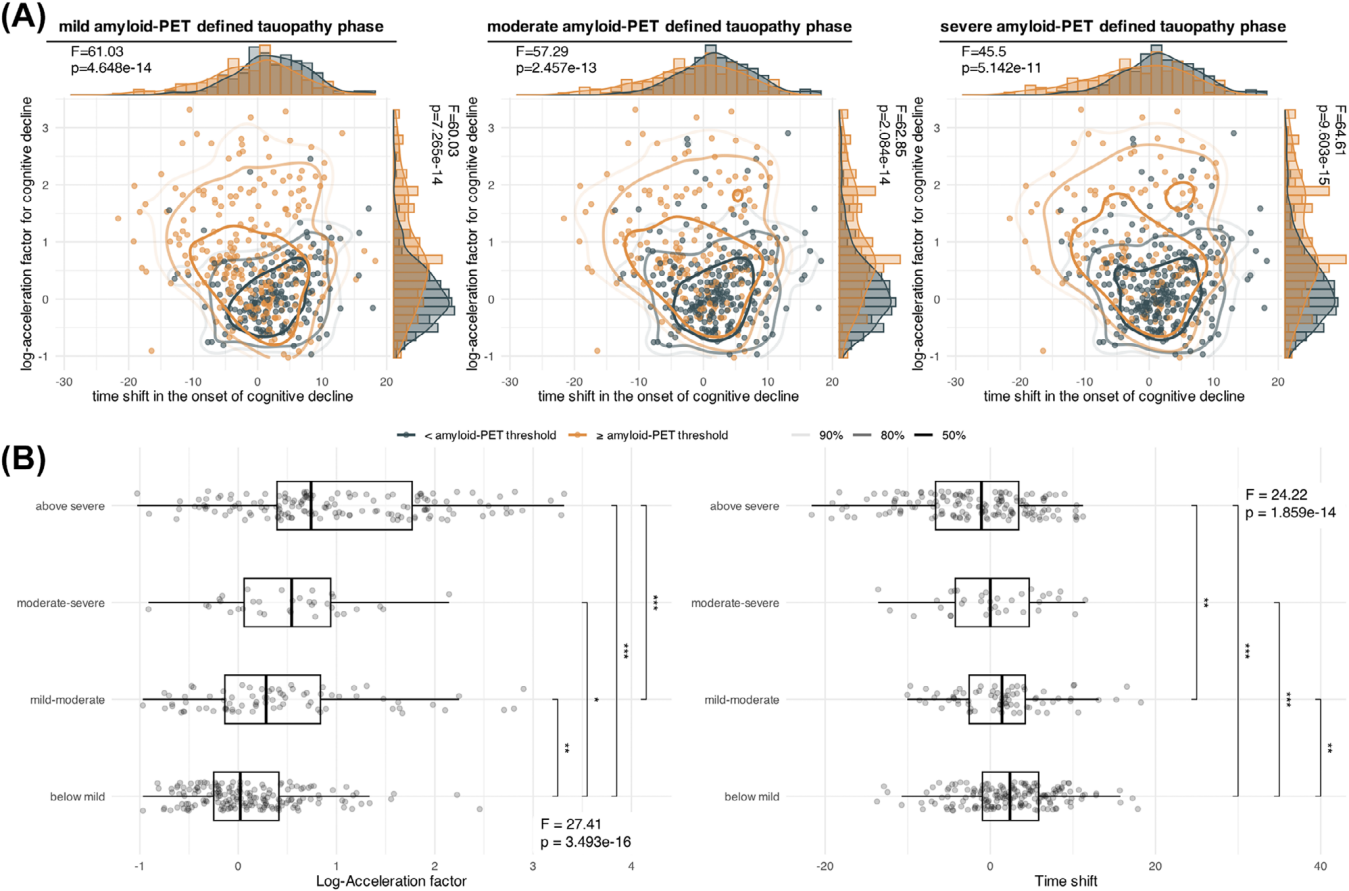
Cognitive decline temporal variability per tauopathy phase status defined by baseline amyloid PET. A, Each dot in the central panel represents each participant's time shift in the onset of cognitive decline compared to the average trajectory and log‐acceleration factor, with distributions shown in the top and right panels. Different colors represent whether participants enter the amyloid PET–defined tauopathy phase. B, Participants were further divided into four groups (i.e., below mild, mild–moderate, moderate–severe, and above severe), followed by ANCOVA and Tukey tests to detect differences in time shift and log‐acceleration factor across each group. Sex, age, education, and cohort were controlled. ^*^ *p* < 0.05, ^**^ *p* < 0.01, ^***^ *p* < 0.001. ANCOVA, analysis of covariance; PET, positron emission tomography.

Third, we determined whether advanced amyloid‐inferred tauopathy was associated with earlier and faster cognitive decline. Therefore, we grouped individuals as falling below/above age‐ and sex‐specific amyloid PET–inferred tauopathy thresholds determined above. Using ANCOVAs, controlling for age, sex, education, and cohort, we found faster cognitive decline in individuals surpassing a given amyloid PET–inferred tauopathy threshold, consistent for MMSE and ADAS‐Cog_11_ (Figure 3A). Similar results were obtained when grouping individuals into gradual groups (i.e., < mild/mild–moderate/moderate–severe/ > severe), where the rate of cognitive decline increased with more advanced amyloid PET–inferred tauopathy stages (Figure 3B). When modeling non‐linear trajectories of cognitive decline, we found that surpassing a given amyloid PET–inferred tauopathy threshold was associated with significantly faster cognitive decline (*p* = 7.265×10^−14^/2.084×10^−14^/9.603×10^−15^ for mild/moderate/severe tauopathy) and an earlier acceleration of cognitive decline (*p* = 4.648×10^−14^/2.457×10^−13^/5.142×10^−14^, Figure 4A), with these effects becoming more evident in more advanced amyloid PET–inferred tauopathy phases (Figure 4B). Together, more advanced stages of amyloid PET–inferred tauopathy translated into more progressive cognitive decline.

## 4 DISCUSSION

Our main goal was to determine patient‐centered amyloid PET thresholds at which amyloid triggers tauopathy. Our first main finding was that younger age was consistently linked to earlier Aβ‐associated tauopathy in men. Additionally, women require less Αβ for transitioning to tauopathy. Second, we translated these findings into age‐ and sex‐specific amyloid PET thresholds at which patients transition from amyloidosis to tauopathy that successfully captured progressive tau deposition and accumulation rates. Third, we showed that exceeding patient‐specific amyloid PET–inferred tauopathy thresholds was associated with accelerated and earlier cognitive decline. Together, these findings suggest that the proposed age‐ and sex‐specific amyloid PET thresholds can help infer a patient's likelihood to transition to tauopathy, without requiring tau PET–based staging. These patient‐centered thresholds may support amyloid PET–based screening for anti‐Aβ treatment, to facilitate Aβ removal prior to tauopathy.

The revised AD criteria treat amyloid PET as a binary measure.^23^, ^57^ While this approach offers a valid indication of Aβ pathology,^57^, ^58^ it does not capture the trajectory of disease progression only by using amyloid PET. Therefore, we propose a set of patient‐centered amyloid PET thresholds designed to identify the onset of Aβ‐related tauopathy. These thresholds complement the binary approach of Aβ presence/absence by reducing the reliance on tau PET for staging and enabling a more individualized assessment of the Aβ–tau relationship.^26^, ^28^, ^29^, ^59^ Caution is still warranted in interpreting the precision of the derived thresholds, and further research with larger and more diverse datasets is needed for refinement and validation. Nonetheless, the conceptual observation remains: differences in amyloid PET cut‐offs may exist across age and sex groups. Specifically, we show that younger age is linked to earlier Aβ‐induced tauopathy, while women transition to tauopathy at lower Aβ. Our findings extend previous studies, which have shown that younger age is linked to stronger Aβ‐related tau accumulation,^28^, ^29^, ^60^ and that women show faster accumulation at a given level of Aβ versus men.^25^, ^26^, ^29^, ^59^, ^60^ Supporting a pronounced susceptibility of women to tau pathophysiology, previous studies found stronger Aβ‐related cerebrospinal fluid (CSF) p‐tau increases in women,^25^, ^61^ which elicit faster tau aggregation in women versus men.^25^ This suggests that women show earlier Aβ‐driven secretion of hyperphosphorylated tau^62^ that promotes tau aggregation. Previous data suggest that endocrine factors may contribute to increased tau vulnerability in women. Animal models indicate that testosterone protects against tau hyperphosphorylation. Therefore, generally lower testosterone levels in women, which drop further after menopause, may promote tau pathophysiology.^63^, ^64^ This is supported by previous work linking lower testosterone to CSF p‐tau increases in individuals at genetic risk of AD.^65^ Beyond endocrine differences, sex differences in neuroinflammation may further contribute to earlier Aβ‐induced tauopathy in women. Specifically, soluble triggering receptor expressed on myeloid cells 2–related neuroinflammation is linked to stronger Aβ‐related p‐tau increases in women versus men^61^ and female AD patients exhibit a heightened neuroinflammatory response on translocator protein PET, which is linked to greater fibrillar tau.^66^ Thus, a combination of endocrine, inflammatory, and potentially other unidentified factors may explain increased susceptibility to Aβ‐related tauopathy in women.

We further confirm age as a key modulator of the Aβ–tau axis, with younger individuals showing an earlier transition to tauopathy. Yet, there was suggestive evidence for variation by sex: (1) in the temporal meta‐ROI, younger age is related to faster Aβ‐related tau accumulation in men, whereas this effect was weaker or absent in women; (2) regionally, a larger number of ROIs exhibited significant age effects in men. Notably, at the global level, women in A05 also exhibited a trend of age effect that is comparable to men. This discrepancy may reflect greater variance in A05 due to the smaller sample size and higher measurement uncertainty.^58^ Overall, our findings align with previous work showing that younger AD patients show faster tau accumulation.^28^, ^29^, ^60^ We found previously that younger AD symptom onset is associated with stronger tau deposition in highly connected cortical hubs,^28^ which amplify tau spread across connected regions.^28^, ^67^, ^68^ Moreover, connectivity declines with aging, especially at hub regions, reducing network efficiency in older adults^69^ and potentially attenuating tau propagation. However, this leaves unexplained why younger age relates to faster Aβ‐related tau accumulation in men but to a lesser extent in women. Potential explanations could be sex‐specific regional tau vulnerabilities or sex differences in the brain's connectome that route tau spread.^70^ Connectome studies reported stronger inter‐network connections in women versus men, which may facilitate tau spreading.^70^ Alternatively, the above‐described endocrine and inflammatory factors promoting earlier Aβ‐associated tau accumulation in women may dominate, blunting age effects. Here, further research is needed to disentangle these possibilities, but our results underscore that age modifies the Aβ–tau axis, potentially in a sex‐related manner.

Translating these observations into patient‐centered biomarker cut‐offs offers practical utility, showing that amyloid PET allows inferring whether a patient has likely entered mild/moderate/severe tauopathy without requiring a tau PET scan. Importantly, individuals who exceeded the amyloid PET–inferred tauopathy cut‐offs showed faster subsequent cognitive decline, consistent with tau pathology being the key driver of cognitive decline in AD.^21^, ^22^, ^71^ In a therapeutic context, this suggests that there may be a critical window before substantial tau accumulation during which anti‐Aβ drugs may be most effective.^13^ By defining the amyloid PET cut‐offs that signal this tipping point for age‐ and sex‐specific subgroups, we provide a tool to potentially guide treatment decisions.

When interpreting our results, several limitations should be considered. First, there were some discrepancies between ADNI and A05: (1) at the global level, women in A05 exhibited a similar trend of age effect comparable to that in men; (2) at the regional level, although effect maps of the two cohorts were partially overlapped, fewer regions in A05 males showed a significant effect (Figure 1B). Given the smaller sample size and higher baseline Centiloid levels (with higher Centiloid values implying greater measurement uncertainty^58^) in A05, together with consistently weaker/absent effects in the temporal meta‐ROI and only limited spatial extensions reaching significance among women participants across cohorts, we infer that these discrepancies likely arise from the limited statistical power and higher variance in A05. Larger datasets will be required for validation. Second, our analyses were based on observational studies and did not include patients from anti‐Aβ trials.^13^, ^14^ Therefore, we cannot directly confirm that treating individuals before versus after crossing the proposed amyloid PET threshold yields differences in clinical outcomes. Prospective validation in anti‐Aβ treatment settings will be important to substantiate the utility of these thresholds in practice. Third, we relied on [^18^F]Flortaucipir to determine fibrillar tau pathology. However, off‐target binding in the meninges is more pronounced in women, which may therefore confound our analyses.^72^ Nonetheless, sex differences in tau pathophysiology have been shown using fluid biomarkers^25^ and *post mortem* data,^73^ hence our findings are unlikely to be fully driven by off‐target binding. Fourth, participants < 65 years old were underrepresented. Therefore, including younger individuals in future studies would help substantiate our findings. Finally, we focused on fibrillar rather than fluid tau markers.^74^ Integrating fluid biomarkers could refine our model by capturing tau pathophysiology in nascent pre‐fibrillar stages,^75^ potentially improving the precision of threshold estimates to pre‐fibrillar tauopathy.

Together, we demonstrate that the transition from amyloidosis to tauopathy in AD is modulated by age and sex. We provide patient‐tailored amyloid PET thresholds linked to tauopathy that could have practical clinical relevance to complement the binary approach, indicating Aβ positivity by reflecting disease progression to tauopathy. This personalized biomarker approach could enhance patient screening, clinical trial design, and inform individualized treatment strategies to combat AD progression.

## CONFLICT OF INTEREST STATEMENT

N.F. received speaker honoraria from GE Healthcare, EISAI, Life Molecular Imaging and consulting honoraria from MSD as well as research support from Eli Lilly. No other authors have any competing interests to declare. Author disclosures are available in the supporting information.

## CONSENT STATEMENT

The ADNI and A05 investigators at each site obtained ethical approval, and all subjects provided written informed consent.

## Supporting information



Supporting Information

Supporting Information
