# Supplementary material for: Defining patient‐centered amyloid PET thresholds for the onset of tauopathy in Alzheimer's disease
Source: Alzheimers Dement. 2026 Jan 4;22(1):e71064. doi: 10.1002/alz.71064 (PMC12765405; doi:10.1002/alz.71064)
Supplement: Supplementary file 1 — Supporting Information [file ALZ-22-e71064-s001.docx]

**
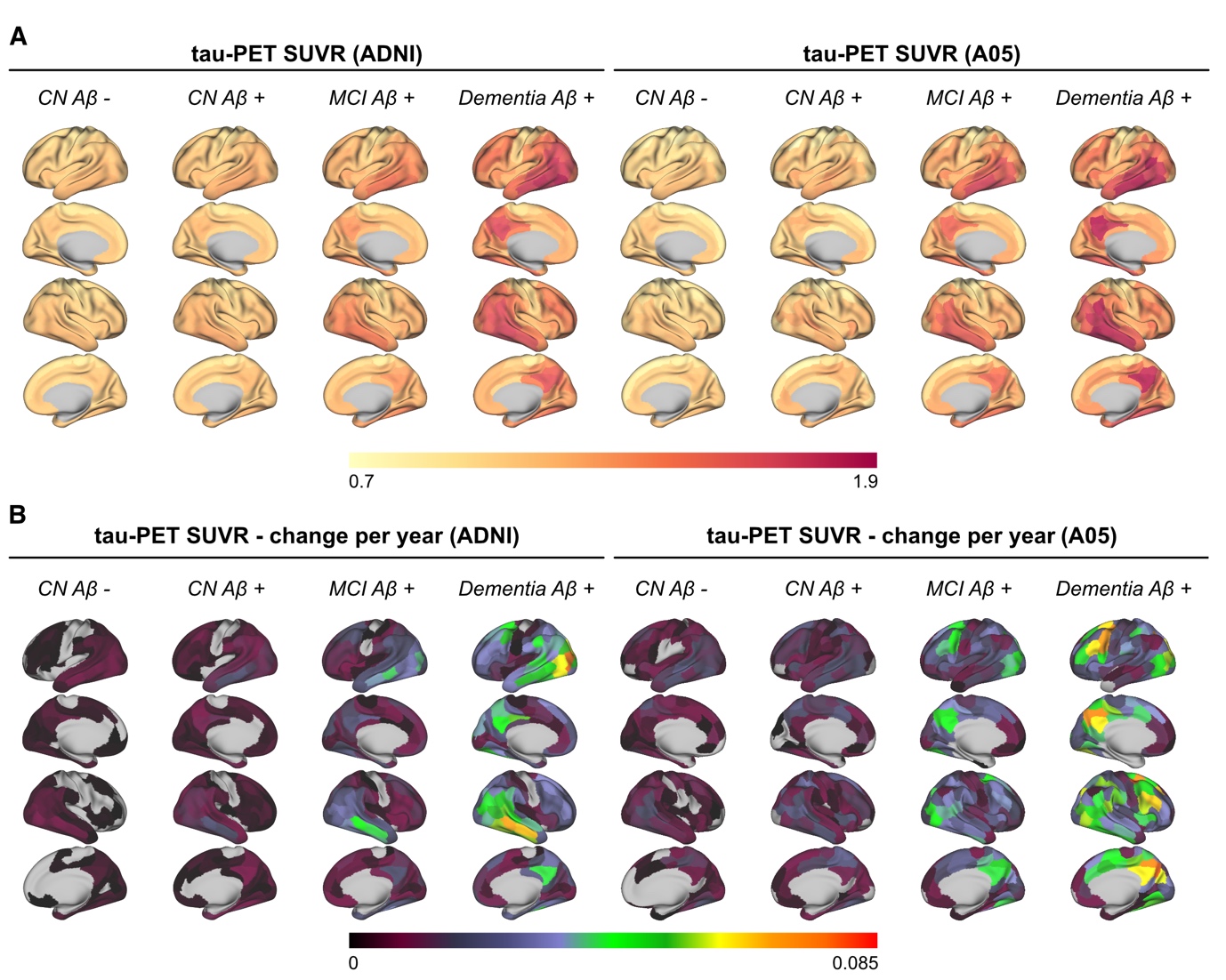
**

**eFigure 1.** Baseline tau-PET (A) and tau-PET rate of change (B) in ADNI (left panel) and A05 (right panel). CN: Cognitively Normal, MCI: Mild Cognitive Impairment.

**
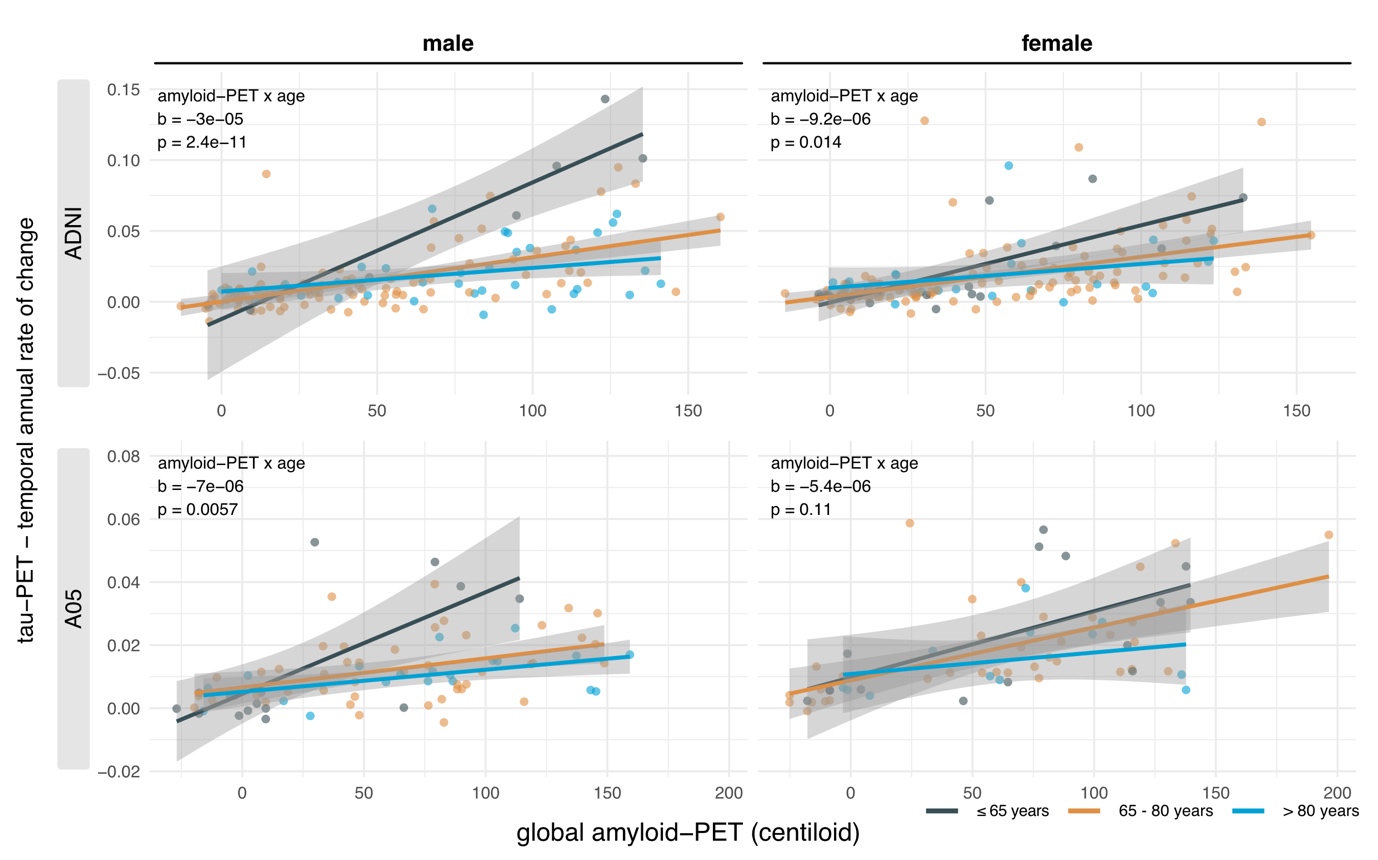
**

**eFigure 2. Younger males are related to faster temporal amyloid-related tau accumulation.** Scatterplots showing the interaction between global amyloid-PET (centiloid) and age on temporal tau-PET annual change rates stratified by sex in ADNI and A05 cohorts.


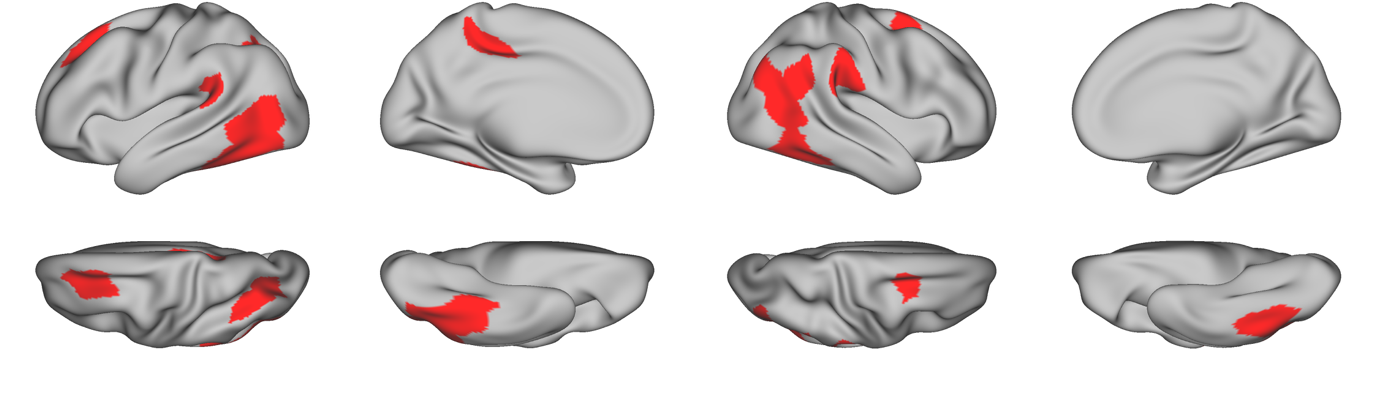


**eFigure 3.** Regions overlapping between ADNI and A05 male data with a significant age x amyloid-PET interaction (FDR-corrected). Statistics were derived from robust regression models controlling for ApoE4 carrier status.

**
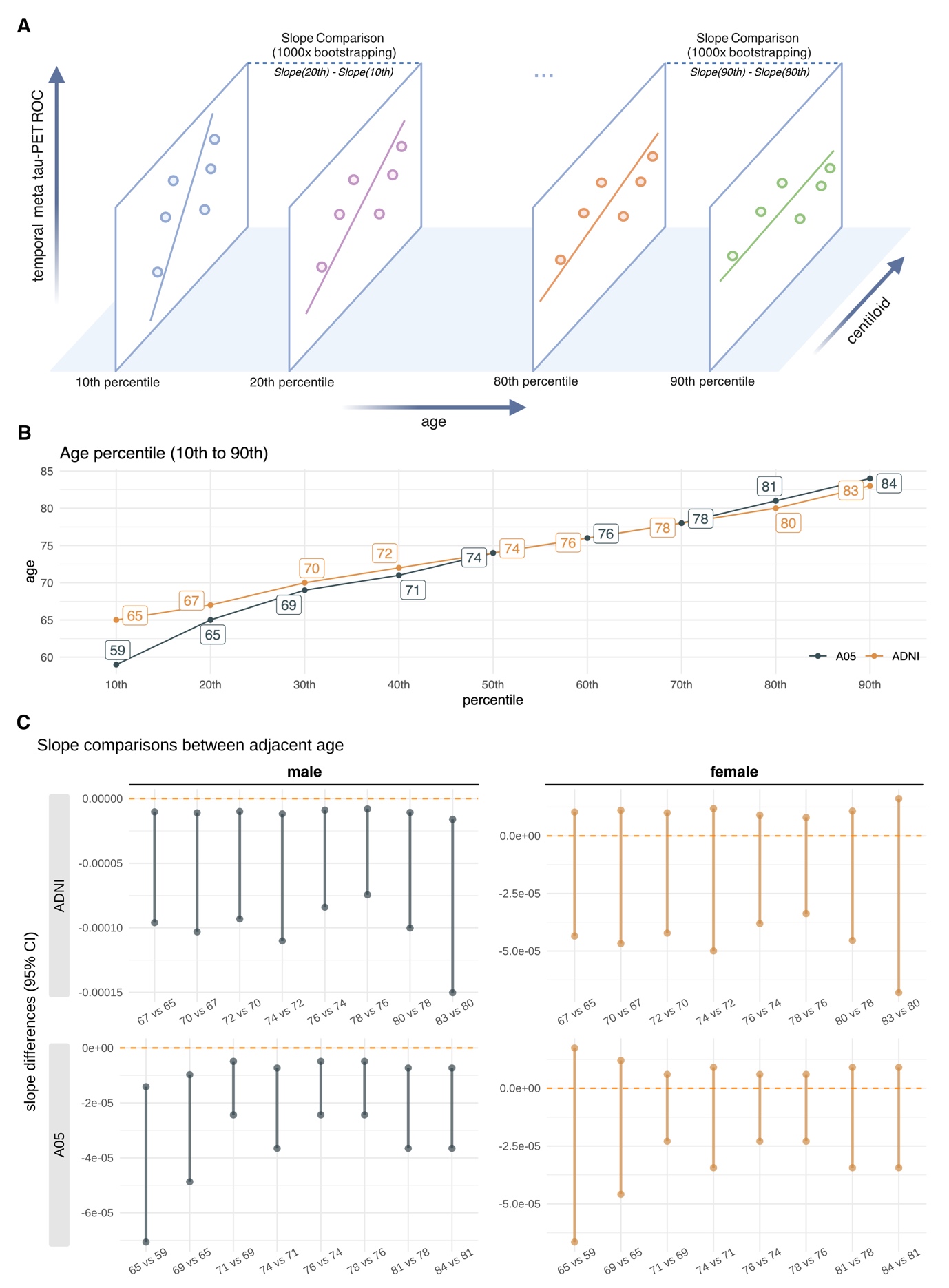
**

**eFigure 4.** **Slope comparisons by age in temporal meta region.** (A) Graphic illustration of slope comparison (image was created with BioRender.com). Robust regression models were constructed in males and females separately. The slopes of amyloid-PET in regression equations were calculated at each of the 10th–90th age percentiles (B). The 1000x bootstrapping method was further used to detect the differences between two adjacent percentiles of age (C). Younger age is consistently associated with faster amyloid-related tau accumulation in males but not in females. Each segment represents the 95% CI of the slope difference between two adjacent age groups, determined by subtracting the slope at the younger age from the slope at the older age. Segments falling below zero indicate that the slope is significantly greater at the younger age compared to the older age.

**eTable 1.** Sample characteristics by cohort

|  | **ADNI** (n = 301) | **A05** (n = 143) | **p-value** |
| --- | --- | --- | --- |
| Sex (male/female) | 140/161 | 80/63 | 0.068 |
| Global amyloid-PET (Centiloid, M/SD) | 49.72 (42.11) | 60.86 (52.13) | 0.027 |
| Age (M/SD) | 74.04 (7.192) | 72.94 (9.383) | 0.217 |
| Global tau-PET ROC (M/SD) | 0.0080 (0.0183) | 0.0190 (0.0214) | <0.001 |
| Temporal tau-PET ROC (M/SD) | 0.0175 (0.0243) | 0.0152 (0.0143) | 0.211 |
| APOE4_status (pos/neg) | 147/154 | 66/77 | 0.613 |
| DX (CN/MCI/Dementia) | 206/64/31 | 44/58/41 | <0.001 |

Two-sample t tests were used for continuous variables, and Fisher's exact tests were used for categorical variables in group comparisons, with a two-sided alpha level of 0.05.

**eTable 2.** Effect of Amyloid-PET x age on global tau-PET ROC (cognitive functional status adjusted)

| Cohort | Sex | B value | p_Bonferroni-corrected_ | Partial R^2^ |
| --- | --- | --- | --- | --- |
| ADNI | Male | -7.87e-06 | 0.038 | 0.0490 |
| ADNI | Female | -1.44e-06 | > 0.9 | 0.00367 |
| A05 | Male | -1.12e-05 | < .001 | 0.258 |
| A05 | Female | -9.38e-06 | 0.081 | 0.0831 |
